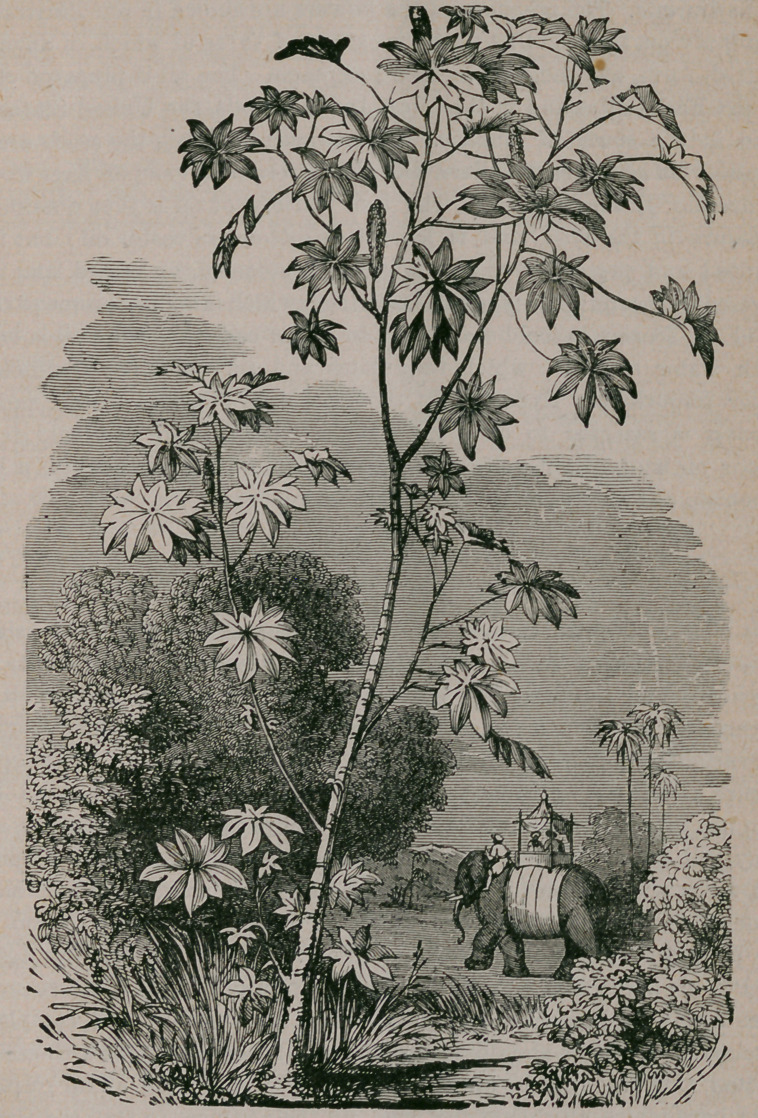# The Castor Oil Plant

**Published:** 1888-02

**Authors:** 


### THE CASTOR OIL PLANT.

The castor oil plant belongs to an order whose affinities have not yet been accurately limited by botanists; but it is supposed to comprise at least one thousand five hundred species, distributed in each quarter of the globe from the equator to latitudes as high as Great Britain; sometimes in the form of large trees, frequently of bushes, still more usually of diminutive weeds, and occasionally of deformed, leafless, succulent plants, resembling the cacti. The plant is highly valuable for the excellent medical virtues of the oil which it furnishes; its root is said to be diuretic. The positions of the flowers are shown in our illustration; but it is from the seed that the oil is extracted, three of which, of an oblong flattish form, are enclosed in each receptacle. The oil is prepared chiefly in the East Indies and in the West India Islands, the United States, and also in the south of Europe. In extracting the oil, the seeds are first bruised between heavy rollers, and then pressed in hempen bags under a hydraulic or screw press. The best variety of oil is thus obtained by pressure in the cold, and is known as *cold-drawn* castor oil; but if the bruised and pressed seeds be afterwards steamed, or heated, and again pressed, a second quality of oil is obtained, which is apt to become partially solid or frozen in cold weather. In either case the crude oil is heated with water to 212°, which coagulates and separates the albumen and other impurities. Exposure to the sun's light bleaches the oil, and this process is resorted to on a large scale. When pure and cold drawn, castor oil is of a light-yellow color, but when of inferior quality, it has a greenish, and occasionally a brownish tinge.

## Figures and Tables

**Figure f1:**